# Is Bilateral Facial Paralysis an Indicator of Respiratory Outcome in Guillain–Barré Syndrome?

**DOI:** 10.3390/medicina55050177

**Published:** 2019-05-21

**Authors:** Serdal Güngör, Betül Kılıç

**Affiliations:** Inönü University, Faculty of Medicine, Department of Pediatric Neurology, 44060 Malatya, Turkey; gungorserdal@yahoo.com

**Keywords:** bilateral facial paralysis, Guillain–Barré syndrome, respiratory outcome

## Abstract

*Background and objectives:* Bilateral facial paralysis is a rare and specific clinical manifestation of various neurological disorders. Bilateral facial paralysis has been reported as an essential feature of Guillain–Barré syndrome (GBS) for many years. We aim to describe the incidence of bilateral facial paralysis and prognosis in our GBS patients. *Materials and Methods:* A retrospective chart review of all patients with GBS and bilateral facial paralysis who were treated at the Inönü University Medical Faculty was performed. *Results:* A total of 45 cases of GBS were reviewed. Four out of 45 patients (8.8%) had associated bilateral facial paralysis. Only one of the patients also had acute multiple cranial neuropathies. All patients experienced sudden deterioration and respiratory distress. In one of our patients who had multiple cranial neuropathies, serum antiganglioside antibody assay was performed, and anti-GQ1b IgG antibody positivity was observed. The cerebrospinal fluid had albuminocytological dissociation in all patients, and axonal involvement was present in nerve conduction studies (NCS). Three patients improved with immunotherapy; one patient died due to cardiac arrest after resistant hypotension. *Conclusion:* Bilateral facial paralysis is a rare condition in children. We wanted to emphasize bilateral facial involvement and poor prognosis in our GBS patients.

## 1. Introduction

Bilateral simultaneous peripheral facial paralysis (BFP) is seen with a frequency rate of less than 1% of patients who have facial paralysis. Since the observed effect on children is very rare, the diagnosis is quite difficult [1]. The underlying causes of most of these cases are severe medical conditions, and in some cases may require an emergency medical treatment. Lyme disease, Leukaemia, Sarcoidosis, Guillain–Barré syndrome (GBS), mononucleosis infections, and trauma are the general causes of bilateral facial paralysis. According to the report, only 20% of these cases are idiopathic or Bell’s palsy. In those cases, there is no proof of systemic or local disease [2,3].

Guillain–Barré syndrome is a disease with various clinical manifestations. Classification is grouped into acute inflammatory demyelinating polyneuropathy and axonal variants (e.g., Miller–Fisher syndrome, Bickerstaff brain stem encephalitis, pharyngo-cervical-brachial variant, pharyngo-cervical-brachial variant, polyneuritis cranialis, and acute sensory neuropathy) [4,5,6,7,8,9].

Here, we aim to describe the incidence of bilateral facial paralysis caused by GBS with the clinical, laboratory, and electrophysiological features and outcomes of patients in our cohort.

## 2. Materials and Methods

We performed a retrospective chart review of all patients with GBS who were treated at Inönü University Medical Faculty between 2009 and 2017. Before accessing the patients’ records, approval was obtained from the Ethical Committee of Clinical Investigations of the Medical Faculty of Inönü University. Informed consents were also obtained from the relatives of the patients for publication of the photographs. Clinical and demographic data were collected, including age, gender, presenting symptoms and predisposing diseases, laboratory findings, electrophysiological features, treatment modalities, and clinical course during follow-up.

## 3. Results

The clinical and laboratory findings of the BFP cohort are summarized in Table 1.

### 3.1. Case 1

**Figure 1:**
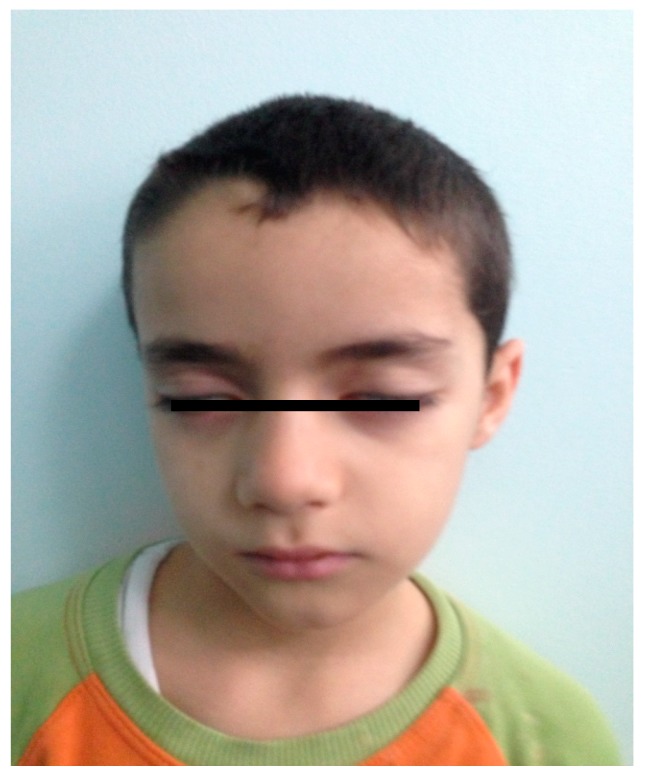
The appearance of bilateral facial paralysis in Case 1.

A seven-year-old male patient applied with an unbalanced walking complaint. Ten days earlier he was diagnosed with hepatitis A and had jaundice. The patient had no other important details in his medical history. Bilateral peripheral facial paralysis and ataxia were detected on physical examination (Figure 1).

Of all muscle groups, lower limb muscle power was 4/5. Bilateral lower extremity deep tendon reflexes were decreased. There was no bowel or bladder involvement.

Blood tests were performed to examine full blood counts, electrolytes and urea, and all were within the reasonable limits. Magnetic Resonance Imaging (MRI) scan of the head did not reveal any pathology. Cerebrospinal fluid (CSF) examination showed a high protein concentration (146.9 mg/dL, standard 45 mg/dL), with no white blood cells. Nerve conduction studies (NCS) revealed marginally undetected tibial F-waves and sensory neuropathy with a decrease of sensory nerve action potentials of the sural and median nerve accompanied. Median and peroneal nerve combined muscle action potential (CMAP) amplitudes were absent. Intravenous immunoglobulin (IVIG) treatment was given with a dose of 2 g/kg for five days. Respiratory distress and hypertension developed on the second day of admission, and the patient had a mechanic ventilation requirement. Plasma exchange was performed every other day, and total of three times. The patient’s facial paralysis and ataxia partially regressed 14 days after onset, and disappeared completely after 23 days. There has been no proof of recurrence one year later.

### 3.2. Case 2

**Figure 2:**
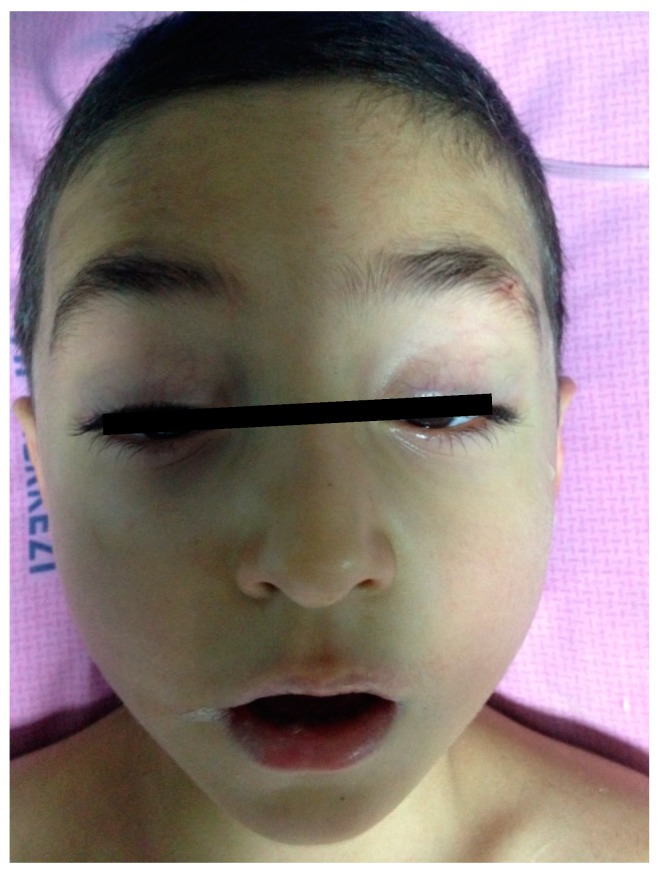
The appearance of bilateral facial paralysis in Case 2.

A six-year-old boy was brought to the emergency department with a complaint of difficulty walking after five days of an upper respiratory tract infection. On the physical examination, bilateral peripheral facial paralysis was seen (Figure 2). Muscle strength of bilateral limbs were 3/5, and arms were 4/5. Deep tendon reflexes on bilateral limbs were hypoactive.

Hemogram, serum biochemistry, and potassium test were regular, serology for antinuclear antibody (ANA), hepatitis B surface antigen (HBsAg), and human immunodeficiency virus (HIV) were negative. In the examination of CSF, CSF protein was 85 mg/dL, and 4/mm^3^ lymphocyte was found in the CSF. In electrophysiological studies, sensory nerve conduction studies were normal. Median, ulnar and peroneal nerve CMAP amplitudes were absent. Posterior tibial nerve CMAP amplitudes were decreased, distal motor latency and motor conduction velocities were normal. IVIG was given with 2 gr/kg/day dose for two days. Three days after the hospitalization, rapid progressive paralysis developed at all four extremities, accompanying respiratory distress and tachycardia. The patient required ventilation support. Plasma exchange was performed every other day, and total of five times due to the poor general condition. Mycoplasma Ig M was positive. The patients’ respiratory distress and paralysis recovered completely after seven days. Since his facial paralysis had also regressed in 14 days after onset, the patient was out of follow-up.

### 3.3. Case 3

**Figure 3:**
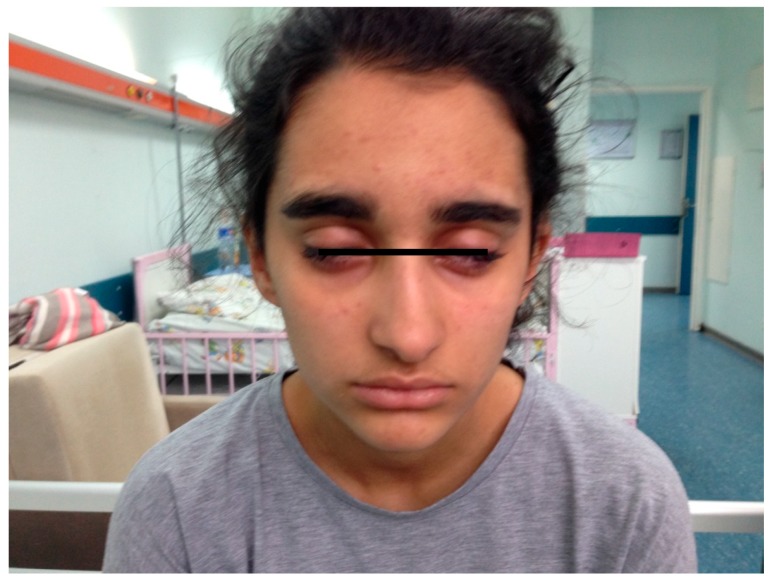
The appearance of bilateral facial paralysis in Case 3.

A 14-year-old female patient was admitted to the children’s neurology department for bilateral peripheral facial paralysis, diplopia, ptosis, dysphagia, and dysarthria with no weaknesses on the limbs (Figure 3). The prodromal symptoms included upper respiratory tract infection for seven days. The patient had no significant details in her medical, neurological or family history.

Bilateral ptosis and mydriasis, slurred speech, difficulty in swallowing, aphonia, bilateral palatal palsy, tongue movement limitation, and hypoacusia occurred on the second day of hospitalization. Sternocleidomastoid and trapezius muscles strengths were weakened. Bilateral muscle strength of arms were 3/5, and limbs were 4/5, but the patient’s tendon reflexes were well protected. Extensively, she indicated bilateral CN III, IV, VI, VII, VIII, IX, X, XI, and XII involvements, but there were no signs of cerebellar involvement such as ataxia, or any other symptoms indicating sphincter or autonomic dysfunctions. The patient fully co-operated and was conscious with no cognitive impairments. Cranial MRI imaging was normal. The laboratory tests including blood count, thyroid function tests, Venereal Disease Research Laboratory (VDRL) and antinuclear antibody (ANA), C3, C4 were normal. On the tenth day of the illness, CSF was tested, and white blood cell count was normal, but total protein concentration (98 mg/dL) was significantly elevated. Due to the rapid progression, respiratory distress, and hypertension, a daily plasma exchange was planned, and applied a total of five times, every other day. NCS showed reduced CMAP amplitudes in motor conductions with normal nerve conduction after three weeks of illness. Serum antiganglioside antibodies, anti-GQ1b were positive while GM1, GD1b, GT1a, GT1b, GM3, and GM2 were negative. As an etiological agent, human rhinovirus was positive. Patients dysarthria and extraocular palsy begin to regress after seven days. Multiple cranial neuropathies of the patient which include facial paralysis entirely recovered on day 52. A follow-up study after 1.5 years did not indicate any proof of recurrence.

### 3.4. Case 4

**Figure 4:**
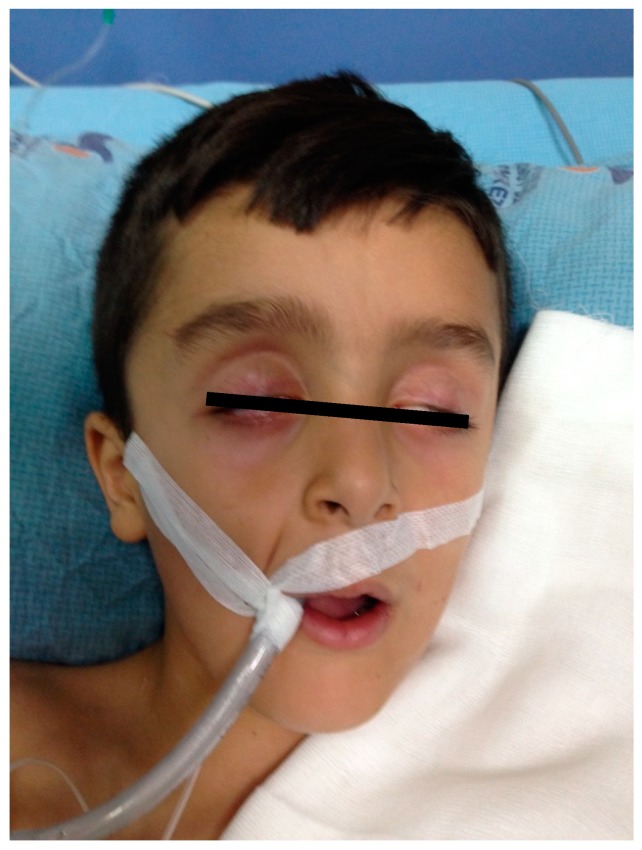
The appearance of bilateral facial paralysis in Case 4.

An eight-year-old boy was brought to the emergency department with sudden onset of weakness and respiratory distress. His consciousness was confused. Areflexic acute flaccid paralysis was present on all four extremities. Laboratory tests showed elevated erythrocyte sedimentation rate in small quantities (erythrocyte sedimentation rate; 21 mm/h, average 15 mm/h). Blood count, acetylcholine receptor antibody, thyroid function tests, and other laboratory findings were in normal range. There was no cell in CSF, but CSF protein was high (65 mg/dL). Bilateral peripheral facial paralysis developed on the second day of hospitalization (Figure 4). NCS showed a decrease of the upper extremity sensory and motor action potential, with increased distal latencies. Bilateral peroneal–tibial nerve combined muscle action potential (CMAP) and sural nerve sensory action potential could not be obtained. Bilateral peripheral facial paralysis developed on the second day of hospitalization (Figure 4). Plasma exchange was given every other day, and total of three times due to the respiratory distress. He developed resistant hypotension as an autonomic dysfunction and pulmonary infections. Hypotension could not be controlled, and the patient developed cardiac arrest and died on the sixth day of hospitalization.

## 4. Discussion

GBS is considered as an acute inflammatory polyradiculoneuropathy, characterized with sudden symmetric, progressive, areflexic flaccid paralysis with albuminocytological dissociation [10]. Guillain–Barré syndrome is not only a single entity, but also an union of a pathologically and clinically heterogeneous group of neuropathic conditions. A group of Guillain–Barré syndrome variances is qualified by regional/localized commitment of the peripheral and autonomic nerves [11]. Recognition of this typical case is critical, hence diagnosis allows the anticipatory monitoring of complications and defines therapeutic options for children who are affected.

In the clinical variants of GBS cranial nerve involvement, mechanical ventilation requirement and autonomic findings were reported at a significant frequency, emphasizing that close monitoring and supportive care is crucial for early detection and treatment of these patients. Lin J et al. emphasized that, especially during atypic clinical manifestations of GBS increase severity of motor disfunction, also cranial nerve involvement is potential need for ventilator support, and must alert health care providers to check respiratory signs. These symptoms are associated with poor prognoses [12]. In GBS variants, facial paralysis was reported at frequency of 50% and is usually a poor prognosis indicator [13]. In recent studies, BFP was considered as a rare regional subtype of classical GBS and found to be a demyelinating-type neuropathy [14].

Multiple cranial neuropathies may appear as variants of GBS. In the literature, it is called “polyneuritis cranialis”, and consists of 3–5% of the variant cases. Patients who are affected present BFP soon after the bulbar dysfunctions (CN IX and CN X) with walking difficulties and weakness of limbs [15].

In our GBS patients, only 8.8% had bilateral facial paralysis, and only one patient (2.2%) had multiple cranial involvements. All of our patients had a poor prognosis and mechanical ventilation requirement. Axonal involvement has been as a poor prognostic indicator in our patients.

Most studies accuse anti-GQ1b antibodies in the pathophysiology of cranial nerve involvement [11,14]. GQ1b ganglioside is improved in human’s paranodal regions of the extramedullary portions of abducens, trochlear, and oculomotor nerves, and weakly stains the deep cerebellar nuclei. Anti-GQ1b antibodies are effective mostly at neuromuscular junctions, and cause massive release of acetylcholine which block neuromuscular transmission from nerve terminals, and ultimately cause a structural breakdown of the motor nerve terminal [10]. Most of the patients who have antibodies against GQ1b also have antibodies against GT1a; thus cross-reactivity is recognized. Anti-GT1a is more specific to the lower cranial and is related to bulbar palsy; therefore, cross-reactions between anti-GQ1b and GT1a may cause bulbar palsy [16]. It is evident that, those cross-reactions among anti-ganglioside antibodies might explain the symptoms which were determined in our case 3.

Steroids are not suitable for GBS treatment. However, both immunotherapy plasma exchange and intravenous immunoglobulin are the advised treatments for the classical and variant forms [12]. Eye care should be recommended to avoid corneal complications. The use of recommended face exercises and electrical therapy in Bell’s palsy are controversial.

## 5. Conclusions

Bilateral facial palsy and multiple cranial neuropathies were reported to occur in some variants of GBS. We defend that the progress of the course is generally more rapid in this variant of GBS, respiratory paralysis may occur, and severe progression may be seen. Therefore, close monitoring of GBS patients, especially those with bilateral facial palsy, is required. Further related studies with larger sample sizes are required to explain the value of CSF IgG, and to understand the etiopathology of the disease, and to define different GBS sub-types.

## Figures and Tables

**Table 1:** Demographic Data, Clinical Characteristics, and Management of 4 Cases.

Case	Age/Sex	Predisposing Disease	Clinical Presentation	CSF Protein Concentration	NCS	Treatment	Recovery Time (day)
1	7/M	Hepatitis A	Bilateral facial paralysis, ataxia, lower limb muscle strength and deep tendon reflexes decreased, respiratory distress, hypertension	146.9 mg/dL	AMSAN	IVIG, PE	23
2	6/M	None	Absence walking, bilateral peripheral facial paralysis, decreased bilateral upper and lower limb muscle strength, deep tendon reflexes at bilateral limbs are hypoactive, respiratory distress, tachycardia	85 mg/dL	AMAN	IVIG, PE	14
3	14/F	Human Rhinovirus	Bilateral facial paralysis, ptosis, diplopia, dysarthria, dysphagia, mydriasis, slurred speech, difficulty in swallowing, aphonia, bilateral palatal palsy, limitations of tongue movements and weakened hearing, decreased bilateral upper and lower limb muscle strength, bilateral deep tendon reflexes normoactive, respiratory distress, hypotension	98 mg/dL	AMAN	IVIG, PE	52
4	8/M	None	Global weakness, respiratory distress, confused consciousness, areflexic acute flaccid paralysis, bilateral facial paralysis, resistant hypotension	65 mg/dL	AMSAN	PE	Exitus

Abbreviations: AMAN: Acute Motor Axonal Neuropathy, AMSAN: Acute Motor and Sensorial Neuropathy, IVIG: Intravenous immunoglobulin, PE: Plasma exchange.
